# Association of frailty and pre-frailty with cardiovascular mortality: a meta-analysis of 26 cohort studies

**DOI:** 10.3389/fpubh.2025.1688014

**Published:** 2025-11-13

**Authors:** Yan Zhao, Yedan Wu, Zhuohui Liu, Aisong Zhu

**Affiliations:** 1 Zhejiang Key Laboratory of Blood-Stasis-Toxin Syndrome, School of Basic Medical Sciences, Zhejiang Chinese Medical University, Hangzhou, China; 2 Zhejiang Engineering Research Center for “Preventive Treatment” Smart Health of Traditional Chinese Medicine, Zhejiang Chinese Medical University, Hangzhou, China

**Keywords:** frailty, pre-frailty, risk stratification, older adults, cardiovascular disease (cvd), public health, meta-analysis

## Abstract

**Objective:**

This meta-analysis evaluated the association of frailty and pre-frailty with cardiovascular mortality in cohort studies. While frailty is a recognized predictor of poor outcomes, the prognostic role of pre-frailty—a critical intermediate stage—remains less clear. We assessed their associations with cardiovascular mortality, explored heterogeneity, and examined the robustness of findings through publication bias analyses.

**Methods:**

Cohort studies published up to 2025 were systematically searched. Pooled hazard ratios (HRs) with 95% confidence intervals (CIs) were estimated using random-effects models. Heterogeneity was assessed using the *I*^2^ statistic. Subgroup analyses and meta-regression were performed to explore sources of heterogeneity, but no single factor fully explained the high variability observed (*I*^2^ > 80%). Publication bias was evaluated using funnel plots and statistical tests, with no significant bias detected.

**Results:**

Twenty-six cohort studies involving over 4 million participants were included. Frailty was significantly associated with higher cardiovascular mortality (HR = 2.11, 95% CI: 1.86–2.40), and pre-frailty also conferred elevated risk (HR = 1.80, 95% CI: 1.46–2.23). Despite substantial heterogeneity (*I*^2^ > 80%), subgroup analyses and meta-regression did not identify a clear source. No publication bias was found.

**Conclusion:**

Frailty and pre-frailty are consistently associated with increased cardiovascular mortality, emphasizing their value for early risk identification and preventive strategies. Given the observational nature and residual heterogeneity, findings should be interpreted cautiously, and future research is needed to establish standardized assessment tools and test targeted interventions.

**Systematic review registration:**

https://www.crd.york.ac.uk/PROSPERO/ identifier CRD420251109559.

## 1 Introduction

Cardiovascular diseases (CVDs) remain the leading cause of mortality worldwide and their burden is expected to rise further with population aging (1, 2). Frailty, a multidimensional syndrome characterized by reduced physiological reserve and increased vulnerability to stressors, is highly prevalent among older adults and often coexists with chronic conditions such as CVD, diabetes, and hypertension (3, 4). Frailty may accelerate adverse cardiovascular outcomes through impairments in neuromuscular, immune, and cardiovascular systems (5). Beyond its established links to disability and all-cause mortality (6–8), accumulating evidence indicates that frailty is also a strong predictor of cardiovascular outcomes. For instance, in a large cohort of 154,696 individuals, frailty was associated with a significantly higher risk of cardiovascular events, independent of traditional risk factors (9).

While previous studies have demonstrated that frailty confers excess risks, substantial gaps remain in understanding its prognostic value for cardiovascular mortality specifically. Existing meta-analyses (10–13) have largely focused on patient subgroups, such as those with acute coronary syndrome, chronic heart failure, or hemodialysis, and have typically assessed all-cause mortality rather than cardiovascular mortality as a primary endpoint. More recent reviews (14, 15) included both frailty and pre-frailty, but were restricted to populations with diabetes, prediabetes, or the general population. Consequently, the prognostic impact of frailty—and especially pre-frailty—on cardiovascular mortality among cardiovascular cohorts remains insufficiently clarified.

Pre-frailty, defined as an intermediate stage preceding frailty, is particularly relevant because it is more common, potentially reversible, and frequently overlooked in risk stratification. Clinical evidence further supports its importance: in patients undergoing cardiac surgery, those classified as pre-frail had a substantially higher risk of readmission within 1 year compared with non-frail patients (16). Such findings highlight pre-frailty as a critical target for early identification and intervention, yet its role in predicting cardiovascular mortality has not been systematically assessed.

To address these gaps, our study integrates 26 prospective cohorts with over 4 million participants worldwide. We examined frailty and pre-frailty separately, established cardiovascular mortality as the primary endpoint, and conducted subgroup and sensitivity analyses to explore potential heterogeneity. This approach provides more comprehensive evidence to inform risk stratification and preventive strategies in older adults and populations at high cardiovascular risk.

## 2 Methods

This meta-analysis was conducted in accordance with the Preferred Reporting Items for Systematic Reviews and Meta-Analyses (PRISMA) guidelines (17). The protocol for this review was pre-registered in the International Prospective Register of Systematic Reviews (PROSPERO), with the registration number CRD420251109559.

### 2.1 Data sources

We systematically searched PubMed, Embase, and the Cochrane Library for cohort studies published from the inception of these databases up to July 18, 2025. In addition, we examined the reference lists of relevant systematic reviews and meta-analyses, as well as grey literature sources (e.g., conference proceedings, dissertations, and trial registries) to minimize publication bias. There were no language restrictions applied. The search strategy incorporated both medical subject headings (MeSH) and relevant keywords. Search terms included “Frailty,” “Frailties,” “Frailness,” “Frailty Syndrome,” “Debility,” “Debilities,” “Cardiovascular death,” “Cardiovascular mortality,” and “Mortality.” Additionally, the reference lists of the studies included in the review were manually checked to identify any relevant trials.

A detailed search strategy for Data Sources is provided in Supplementary Table S1.

### 2.2 Eligibility criteria

Studies were included if they met the following criteria: (1) observational study design, (2) exposure factors related to frailty, including both frailty and pre-frailty as defined by each study’s operational criteria (e.g., phenotype, index, checklist, or electronic indices); (3) the outcome of interest was cardiovascular mortality, and (4) studies provided estimates such as odds ratios (OR), relative risks (RR), hazard ratios (HR), along with their corresponding 95% confidence intervals (CI). Studies were excluded if they were meeting abstracts, study protocols, or duplicate publications.

### 2.3 Study selection

The literature was imported into NoteExpress 4.0 for automatic duplicate removal, supplemented by manual checking. For studies with overlapping cohorts, we included the report with the largest sample size or the longest follow-up duration. If overlapping analyses were based on large databases (e.g., NHANES) but examined distinct populations, they were considered independent studies and included. Two reviewers (ZY and WYD) independently screened the titles and abstracts to exclude duplicates and irrelevant articles. Full texts of potentially eligible articles were then reviewed to identify suitable studies. Disagreements were resolved by a third reviewer (LZH).

### 2.4 Data extraction

Data extraction was independently performed by two reviewers (ZY and WYD) following established guidelines for systematic reviews and meta-analyses (18). Data were extracted using pre-designed forms, which included the following information: first author, year of publication, study design, country of origin, population characteristics, study period, sample size, frailty classification, criteria for cardiovascular death, and adjusted confounders. Any discrepancies were resolved through discussion with LZH, and consensus was reached.

### 2.5 Risk of Bias

The risk of bias in the included cohort studies was assessed using the Newcastle-Ottawa Scale (NOS) (19). The NOS assigns a star rating to each cohort study, ranging from 0 to 9. It evaluates three domains: selection of participants (up to 4 stars), comparability of groups (up to 2 stars), and outcome assessment and follow-up (up to 3 stars). Studies with scores of 0–3, 4–6, and 7–9 were classified as low, medium, and high quality, respectively.

### 2.6 Statistical analysis

We calculated the adjusted Hazard Ratios (HR) and 95% confidence intervals (CI) for each study to assess the association between frailty status and cardiovascular death. The heterogeneity of the studies was assessed using the *χ*2 test and the *I*^2^ statistic. If *p* > 0.1 and *I*^2^ ≤ 50%, a fixed-effects model was applied; otherwise, a random-effects model was used. When substantial heterogeneity was present (*I*^2^ > 80%), additional subgroup analyses and meta-regression were conducted to further explore potential sources. Sensitivity analysis was performed by sequentially removing one study at a time to test the robustness of the overall effect. Funnel plots were visually inspected to evaluate publication bias, and Egger’s regression test was used for statistical assessment. With 26 studies included, the test was considered sufficiently powered according to current methodological recommendations (>10 studies).

Subgroup analyses were prespecified by sex, study location, population characteristics, and frailty status (including pre-frailty). To further explore heterogeneity, additional subgroup analyses and meta-regression were performed according to: (1) frailty definition (phenotype-based vs. deficit accumulation); (2) mean age (continuous and stratified); (3) follow-up duration (short vs. long); (4) study design (prospective vs. retrospective); (5) underlying population type (CVD, metabolic/renal, dialysis, or general cohorts); and (6) definition of cardiovascular mortality (ranging from narrowly defined causes such as AMI, SCD, malignant arrhythmias, or HF death to broader ICD-10 I00–I99 classifications). All statistical analyses were performed using Stata version 18 (Stata Corp, College Station, TX).

## 3 Results

### 3.1 Literature search

**Figure 1:**
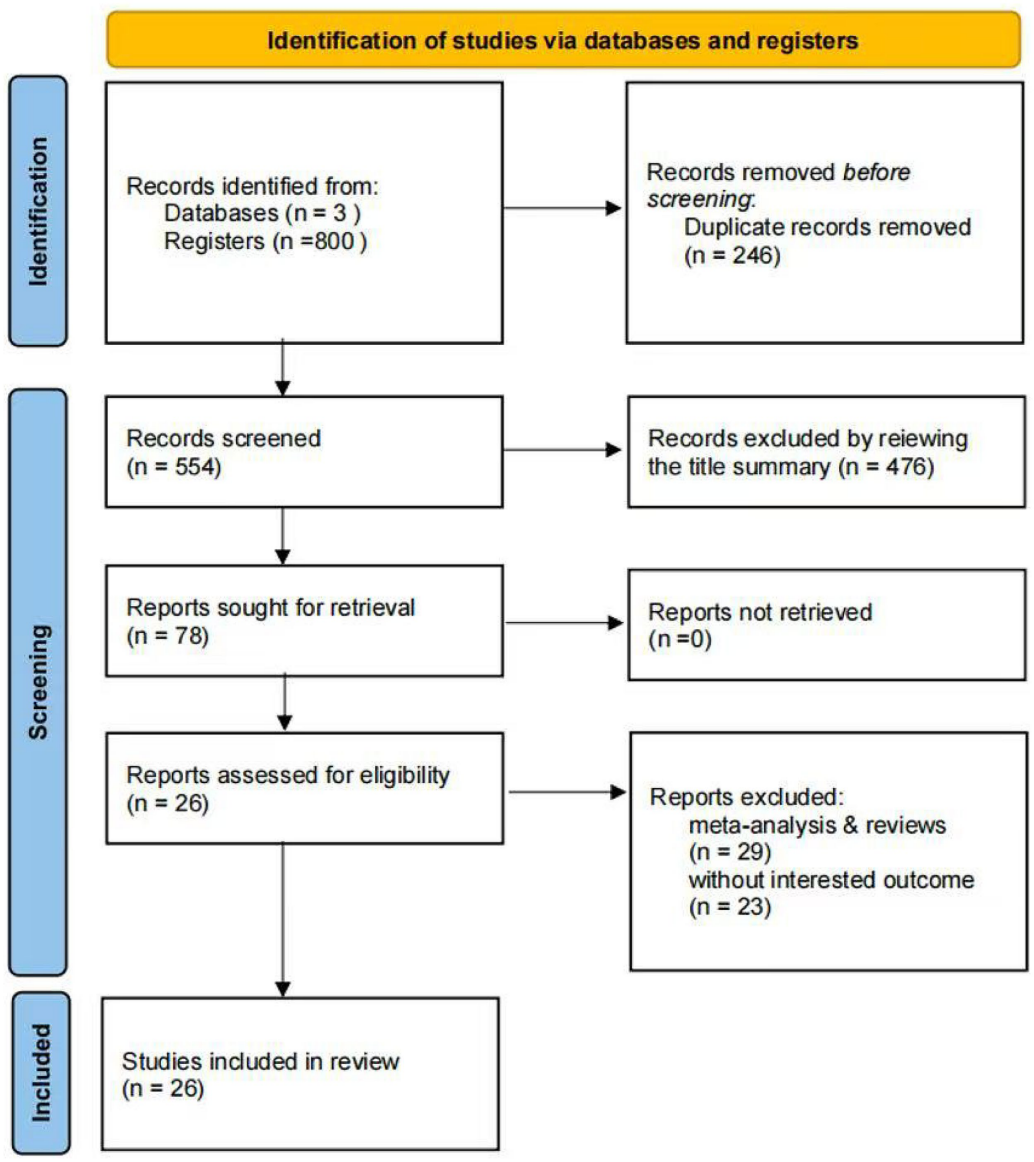
PRISMA 2020 flow diagram of study selection. A total of 800 records were identified; after removing duplicates and irrelevant studies, 26 articles were included in the meta-analysis.

A comprehensive systematic search was conducted for cohort studies published before July 18, 2025. Initially, 800 records were identified. After screening titles and abstracts, 246 duplicate articles and 29 meta-analyses or reviews were excluded. Subsequently, 49 articles were deemed potentially relevant. Upon reviewing the full texts, 26 studies met the inclusion criteria and were included in this meta-analysis. The selection process is illustrated in Figure 1.

### 3.2 Study characteristics

This meta-analysis incorporated 26 cohort studies involving 4,049,963 individuals from diverse geographical regions, with the majority of studies conducted in North America, Asia, and Europe. The studies were published between 2015 and 2025. Of the included studies, 11 were prospective cohort studies, while the remaining were retrospective. Regarding population type, 11 studies were community-based, while the others focused on disease-specific cohorts: four in atrial fibrillation, three in heart failure, four in diabetes or prediabetes, two in chronic kidney disease or dialysis, and two in myocardial infarction or angina. Follow-up ranged from 1 to 20 years; nine studies had ≤2 years of follow-up and four exceeded 10 years. The age distribution also varied. Across studies, the reported mean or median age ranged widely: four studies included populations <65 years, 11 enrolled those ≥75 years, and 5 focused on very old adults (≥80 years). Frailty definitions were heterogeneous: 12 studies used a deficit accumulation approach, the remainder phenotype-based classification. Frailty was dichotomized in 12 studies, while others applied 3–5 severity categories; pre-frailty was specifically assessed in 10 studies. Cardiovascular mortality definitions also varied: five studies restricted outcomes to direct cardiac causes, nine used broader cardiovascular definitions (including stroke and peripheral vascular disease), and three did not specify criteria. All studies, except one that did not specify, adjusted for various confounding factors, which included demographic and clinical characteristics such as age, sex, comorbidities, and lifestyle factors. The main characteristics of the included studies are summarized in Table 1.

**Table 1:** Basic characteristics of the included studies.

Author (year)	Country	Study type	Disease	Age (years)	Follow-up years	Frailty severity	CVD Death criteria	Sample size (CVD deaths size)		Adjusted confounders
Chen et al. (10)	China	Prospective cohort study	MHD	66.6 ± 13.9	Median Follow-up 3.25 years (IQR: 2.4–4)	Modified Fried frailty criteria non-frail FI ≤ 2 frail FI ≥ 3	CAD, PAD, stroke, HF, or AF	Total Non-frail Frail	1,136 (188) 747 (42) 389 (37)	Age, sex, marital status, education, smoking status, hypertension, DM, hyperlipidemia, HF, CAD, PAD, stroke, AF, BMI, BP, Hb, Alb, TC, potassium, calcium, phosphate, dialysis vintage, fluid removal, urea clearance, dialysis frequency
Liu et al. (20)	USA	Retrospective cohort study	NR	69.06 (SE = 0.20)	Median Follow-up 6.5 years (IQR: 5.6–7.6)	FI (49-item) frailty index frailty FI ≥ 0.21	ICD-10: I00–I09, I11, I13, I20–I51	Total Non-frail Frail	2,442 (167) 1,676 (69) 766 (98)	Age, sex, ethnicity, education, marital status, PIR, smoking status, alcohol consumption, physical activity, hypertension, DM, ASCVD, BMI, Alb, UA*, TC
Zhao and Wang (33)	USA	Retrospective cohort study	PreDM	62.89 ± 0.21 (weighted)	Median Follow-up 7.5 years (IQR: 3.8–11.5)	FI (49-item) frailty index frailty FI ≥ 0.21	ICD-10: I00–I09, I11, I13, I20–I51	Total Non-frail Frail	7,845 (636) 5,512 (NR) 2,333 (NR)	Age, sex, education, marital status, PIR, smoking status, alcohol consumption, physical activity, TC, TG, HDL
Gao et al. (22)	China	Prospective cohort study	NR	85 ± 10.1	Median Follow-up 4.03 years (95% CI: 4.02–4.05)	FI (49-item) non-frail FI < 0.25 frail FI ≥ 0.25	ICD-10: I20–I25, I60–I69, I50, I11	Total Non-frail Frail	5,084(280) 1,113 (NR) 3,971(NR)	age, sex, ethnicity, residence, co-residence, education, total income, marital status, BMI, smoking status, alcohol consumption, exercise, physical labor, social activities, pension, fruit intake, vegetable intake, edible oil intake, meat intake
Court et al. (23)	Czech Republic, Poland, Lithuania	Prospective cohort study	NR	59 ± 7.3	13 years (10.9–15.7) Mean (range)	CGA-FI (39-items) non-frail FI < 0.15 Pre-frail 0.15 ≤ FI < 0.25 Mild frail 0.25 ≤ FI < 0.35 Moderate frail 0.35 ≤ FI < 0.45 Severe/Advanced frailty FI ≥ 0.45	ICD-10: I00–I99	Total Non-frail Pre-frail Mild Moderate Severe	14,287 (985) 10,556 (482) 2,840 (310) 612 (98) 165 (39) 114 (29)	Age, sex, country, occupation, education, deprivation level, smoking status, alcohol consumption, physical activity, inverse probability weighting
Tian et al. (30)	USA	Retrospective cohort study	HF	67.3 ± 12.3	Median Follow-up 3.6 years	Cumulative Deficit Model FI (32-items) Non-FI ≤0.210 Moderately frail 0.211–0.310 Severely frail ≥0.311	ICD-10: I00–I09, I11, I13, I20–I51	Total Non-frail Moderate Severe	958 (135) 174 (15) 284 (37) 500 (83)	Age, race, CCI, SBP, eGFR, Alb, UA*
Xiong et al. (21)	USA	Retrospective cohort study	DM	47.6 ± 19.4	Longest follow-up 20 years	FI (49-item) frailty FI ≥ 0.21	ICD-10: I00–I09, I11, I13, I20–I51, I60–I69	Total Non-frail frail	57,098 (2176) 44,491 (1028) 12,607 (1148)	Age, sex, education, poverty, smoking status, alcohol consumption, hypertension, hyperlipidemia, BMI, waist circumference, fasting insulin, glucose, HbA1c, eGFR, creatinine, total bilirubin
Hamada et al. (28)	Japan	Prospective cohort study	HF	81 (IQR:72–87)	2 years	Kihon Checklist (KCL) non-frail 0–3 prefrail 4–7 Frail ≥ 8	SCD, death from worsening HF, death from AMI, and death from CVD and stroke	Total Non-frail Pre-frail frail	936 (113) 145 (2) 290 (23) 501 (88)	Age, sex, SBP, BNP, sodium, eGFR, Hb, EF, use of RAS blockers, beta-blockers
Ohashi et al. (29)	Japan	Prospective cohort study	HF	81(Median)	2 years	Multidomain Frailty FRAGILE-HF FD 0–1 FD 2 FD 3	HF death, ACS, SCD, stroke death, renal death, other CV deaths	Total Non-frail frail	1,181 (133) 530 (51) 651 (82)	Age, sex
Dent et al. (26)	Australia	Prospective cohort study	NR	75.1 ± 2.7	Median Follow-up 12.6 years±3.3	Rockwood FI (49-item) fit FI ≤ 0.12 mildly frail 0.12 < FI ≤ 0.24 frail 0.24 < FI ≤ 0.36 severely frail FI > 0.36	ICD-9: 390–459 ICD-10:I00–I99	Total Non-frail Mild Moderate Severe	1,261 (190) 713 (82) 350 (56) 163 (42) 35 (10)	Age, socioeconomic status, smoking history, physical activity, BMI, plasma 25-hydroxy vitamin D, treatment group, season of blood sampling, prevalent falls, prevalent fractures
Wang et al. (27)	China	Prospective cohort study	NR	80 (IQR:73–87)	4 years	Study of Osteoporotic Fractures Robustness 0 Prefrailty 1, Frailty 2, 3	ICD-10: I00–I99	Total Sustained Non-frail Sustained Pre/Frailty Non-frail to Pre/frail Pre/frail to Non-frail	2,805 (170) 1,043 (41) 832 (75) 498 (36) 432 (18)	Age, sex, education, marital status, income, residence, living with family, smoking status, alcohol consumption, physical activity, regular intake of food, comorbidities, ADL disability
Zhang et al. (34)	USA	Retrospective cohort study	NR	69.5 ± 6.8 (weighted)	Median Follow-up 7.9 years	Fried frailty phenotype	ICD-10: I00–09, I11, I13, I20–51	Total Non-frail frail	6,406 (468) 5,954 (407) 452 (61)	Age, sex, ethnicity, marital status, education, smoking status, depression, hypertension, DM, cardiovascular disease, cancer, BMI
Qin and Zheng (35)	USA	Retrospective cohort study	DM	65.43 (SE = 0.30)	Median Follow-up 6.75 years	FI (49-item) frail FI ≥ 0.21	ICD-10: I00–I09, I11, I13, I20–I51, I60–I69	Total Non-frail frail	2,894 (NR) 1,668 (NR) 1,226 (NR)	Age, sex, race, education, smoking status, alcohol consumption, DM, CHF, obesity, SBP, HDL, Alb, glucose, eGFR, use of anti-diabetic drugs
Hannan et al. (31)	USA	Prospective cohort study	CKD	62.0 ± 10.5	Median Follow-up 12.5 years	Fried frailty phenotype non-frail 0 Pre-frail 1–2 Frail 3–5	MI, CHD, CHF, another CV cause	Total Non-frail Pre-frail frail	2,539 (132) 939 (20) 1,296 (81) 304 (31)	Age, sex, DM, eGFR
Wilkinson et al. (37)	UK	Retrospective cohort study	NR	CKD: 77.5 ± 9.7 no-CKD: 61 ± 12.1	Median Follow-up 5.3 years (IQR: 1.3–5.4)	electronic FI cumulative deficit model non-frail ≤4 mild frailty 5–8 moderate frailty 9–12 severe frailty ≥13	NR	Total Non-frail Mild frail Moderate Severe	819,893 (NR) 405,675 (NR) 308,851 (NR) 85,193 (NR) 20,174 (NR)	Age, sex, ethnicity, social deprivation
Akishita et al. (36)	Japan	Prospective cohort study	NVAF	81.0 ± 4.7	2 years	Kihon Checklist (KCL) non-frail 0–3 prefrail 4–7 Frail ≥ 8	NR	Total Non-frail Pre-frail frail	2,951 (74) 959 (8) 924 (14) 1,068 (52)	Age, sex, BMI, history of major bleeding, AF type, hypertension, severe hepatic dysfunction, DM, hyperuricemia, HF, MI, stroke, thromboembolism, cancer, lipid metabolism disorder, dementia, CrCl, anticoagulant use, falls within 1 year
Shrauner et al. (38)	USA	Retrospective cohort study	NR	76.0 ± 8.3 (2014)	2 years	Cumulative Deficit Model FI (31-item) Non-frail FI < 0.1 Pre-frail 0.1 ≤ FI ≤ 0.2 Mildly frail 0.2 < FI ≤ 0.3 Moderately frail 0.3 < FI ≤ 0.4 Severely frail FI > 0.4	ICD-10: I10–I16, I20–I25, I27–I28, I34-I37, I42, I44-I51, I60–I75, I77–I78, I97, I99, R58, G45, R00	Total	3,068,439 (NR)	Age, sex, race, region, smoking status, hyperlipidemia, statin use, antihypertensive medication use
Nguyen et al. (39)	Asia, Australasia, Europe, North America	Retrospective cohort study	DM	65.8 ± 6.4	4.3 years averages	Rockwood FI (34-item) frail FI > 0.21	NR	Total Non-frail frail	11,140 (NR) 8,275 (NR) 2,865 (NR)	Age, sex, intensive glucose treatment
Liu et al. (40)	USA	Retrospective cohort study	NR	71.0 ± 7.7	Median Follow-up 9.91 years (IQR: 7.58–11.3)	Fried Frailty Phenotype(4) frailty ≥3 pre-frailty 1–2 robust 0	ICD-10: I00–I09, I11, I13, I20–I51, I60–I69	Total Non-frail Pre-frail frail	2,455 (241) 1,692 (NR) 668 (NR) 95 (NR)	Age, sex, race, education, smoking status, DM, hypertension, comorbidities, BMI
Crow et al. (42)	USA	Retrospective cohort study	NR	71.1 ± 0.19	Median Follow-up 8 years (IQR: 6.5–10.3)	Fried Frailty Phenotype(5) frailty ≥3 pre-frailty 1–2 robust 0	ICD-10: I00–I09, I11, I13, I20–I51, I60–I69	Total Non-frail Pre-frail frail	4,984 (521) 2,246 (NR) 2,195 (NR) 541 (NR)	Age, sex, race, education, smoking status, DM, HF, cancer, CAD, arthritis
Kim et al. (43)	Korea	Retrospective cohort study	AF	79.4averages	Median Follow-up 1.9 years (IQR: 0.7–3.5)	comprehensive geriatric assessment FI Robust <0.2 Pre-frail ≥ 0.2 & < 0.35 Frail ≥ 0.35	ICD-10: I20-I21, I25, I50, I10-I12, I34-I35, I38, I46, I61, I63, I69	Total Non-frail Pre-frail frail	365 (48) 121 (6) 68 (5) 176 (37)	Age, sex, CHF, hypertension, DM, stroke, transient ischemic attack, vascular disease, antithrombotic therapy
White et al. (44)	52 countries	Retrospective cohort study	UA/NSTEMI	Non-frail:73(IQR:68–78) frail:75 (IQR:71–81)	Median Follow-up 1.43 years (IQR: 0.87–2.03)	Fried Frailty Phenotype (5) frailty ≥3 pre-frailty 1–2 robust 0	AMI, HF, malignant arrhythmias (e.g., VF, CA), other direct cardiac causes	Total Non-frail Pre-frail frail	4,996 (492) 3,612 (317) 1,147 (137) 237 (38)	Age, region, heart rate, SBP, Killip classification, diuretic use, creatinine, renal insufficiency, ST-segment deviation, troponin elevation, cardiac arrest at admission, previous PCI or CABG, medication, weight
Park et al. (25)	Korea	Retrospective cohort study	AF	67 (IQR:59.5–74.5)	Median follow-up 7.2 years±1.5	Hospital frailty risk score no frailty <5 Frail ≥ 5	ICD-10: I05–I13, I20–I28, I30–I51, I60–I69, I70–I74, I77, I80, I82	Total Non-frail frail	11,953 (1865) 8,729 (716) 3,224 (1149)	Age, sex, HF, hypertension, DM, stroke, MI, vascular disease, osteoporosis, dyslipidemia
Fawzy et al. (24)	French	Retrospective cohort study	AF	77.1 ± 12.1	Median Follow-up 1.1 years	Charlson index ≥4 Frailty index ≥8	ICD-10: I00-I99	total Non-frail frail	12,688 (950) 7,325 (532) 5,363 (418)	Age, sex, CHA2DS2-VASc score
Yu et al. (32)	Russia	Prospective cohort study	STEMI / NSTEMI	77.3 (Median)	1 year	《Age is not a hindrance》 Non-frail 0–2 frail ≥3	Fatal recurrent MI, ACVA, decompensated CHF	Total Non-frail frail	92 (19) 46 (1) 46 (18)	NR
Adabag et al. (41)	USA	Prospective cohort study	NR	76.4 ± 5.6	9.2 years±3.0 (mean±SD)	Fried -Cardiovascular Health Study frail ≥3	ICD-9: 394.9, 396.9–442, 443.9, 459.7, 459.9, 785.51, 996.71	Total Non-frail Intermediate stage frail	3,135 (445) 943 (81) 1717 (242) 475 (122)	Smoking status, stroke, DM, hypertension, CAD, PAD, valvular heart disease, CHF, COPD

Summary of study design, country, sample size, participants’ age, frailty assessment tools, follow-up duration, and reported outcomes. BP, Blood pressure; SBP, systolic blood pressure, DM, diabetes mellitus, CAD, coronary artery disease, PAD, peripheral artery disease; HF, heart failure; AF, atrial fibrillation; CHF, cerebrovascular disease/stroke (stroke), congestive heart failure; MI, myocardial infarction; COPD, chronic obstructive pulmonary disease; HDL, high-density lipoprotein; LDL, low-density lipoprotein; TC, total cholesterol; TG, triglycerides, BMI, body mass index; eGFR, estimated glomerular filtration rate; Hb, hemoglobin; Alb, albumin; UA*, uric acid; hypertension, high blood pressure, glucose, fasting blood glucose/glucose; HbA1c, hemoglobin A1c; BNP, B-type natriuretic peptide, EF, left ventricular ejection fraction, CrCl, creatinine clearance, ASCVD, atherosclerotic cardiovascular disease, CCI, Charlson Comorbidity Index, PIR, poverty-income ratio, and ADL, activities of daily living. MHD, maintenance hemodialysis; CKD, chronic kidney disease; NVAF, non-valvular atrial fibrillation; UA, unstable angina, NSTEMI, Non-ST Elevation Myocardial Infarction; STEMI, ST Elevation Myocardial Infarction; AMI, Acute myocardial infarction; ACS, Acute coronary syndrome; SCD, Sudden cardiac death; CV, cardiovascular; CHD, coronary heart disease; CHF, congestive heart failure; VF, ventricular fibrillation; CA, cardiac arrest; ACVA, acute cerebrovascular accident.

### 3.3 Quality assessment

According to the Newcastle-Ottawa Scale (NOS), the methodological quality of the included studies was generally moderate to high, with an average score of 5.59. Specifically, nine studies (34.6%) were rated as high quality (≥7), 14 (53.8%) as moderate quality (5, 6), and only 3 (11.5%) as low quality (≤4). These findings suggest that most of the included evidence was of acceptable quality, supporting the reliability of the pooled results. The detailed quality scores of the included cohort studies are provided in Table 2.

**Table 2:** The quality assessment of cohort studies.

Study	Year	Selection	Comparability	Outcome	Total
Chen et al. (10)	2025	★★★	★	★★★	7
Liu et al. (20)	2025	★★★	★	★★★	7
Zhao and Wang (33)	2023	★★	/	★★★	5
Gao et al. (22)	2024	★★	/	★★	4
Court et al. (23)	2024	★★★	/	★★	5
Tian et al. (30)	2024	★★★	★	★	5
Xiong et al. (21)	2025	★★★★	/	★★	6
Hamada et al. (28)	2024	★★★	★	★★★	7
Ohashi et al. (29)	2024	★★★	/	★★	5
Dent et al. (26)	2024	★★★★	★	★★★	8
Wang et al. (27)	2024	★★★	★	★★	6
Zhang et al. (34)	2023	★★★	★	★★★	7
Qin and Zheng (35)	2023	★★★	/	★★	5
Hannan et al. (31)	2024	★★★	★	★★	6
Wilkinson et al. (37)	2022	★★★	/	★★	5
Akishita et al. (36)	2022	★★★	★	/	4
Shrauner et al. (38)	2022	★★★	/	★★	5
Nguyen et al. (39)	2021	★★★★	/	★★	6
Liu et al. (40)	2019	★★★	★	★★★	7
Crow et al. (42)	2018	★★★	★	★★	6
Kim et al. (43)	2017	★★★	★	★★★	7
White et al. (44)	2016	★★	/	★★★	5
Park et al. (25)	2024	★★★	★	★★★	7
Fawzy et al. (24)	2025	★★★	/	★★	5
Yu A O	2023	★★★	/	★	4
Adabag et al. (41)	2018	★★★	★	★★★	7

Quality of included studies was assessed using the Newcastle–Ottawa Scale (NOS), with scores provided for selection, comparability, and outcome domains.

### 3.4 Frailty and the risk of cardiovascular mortality

**Figure 2:**
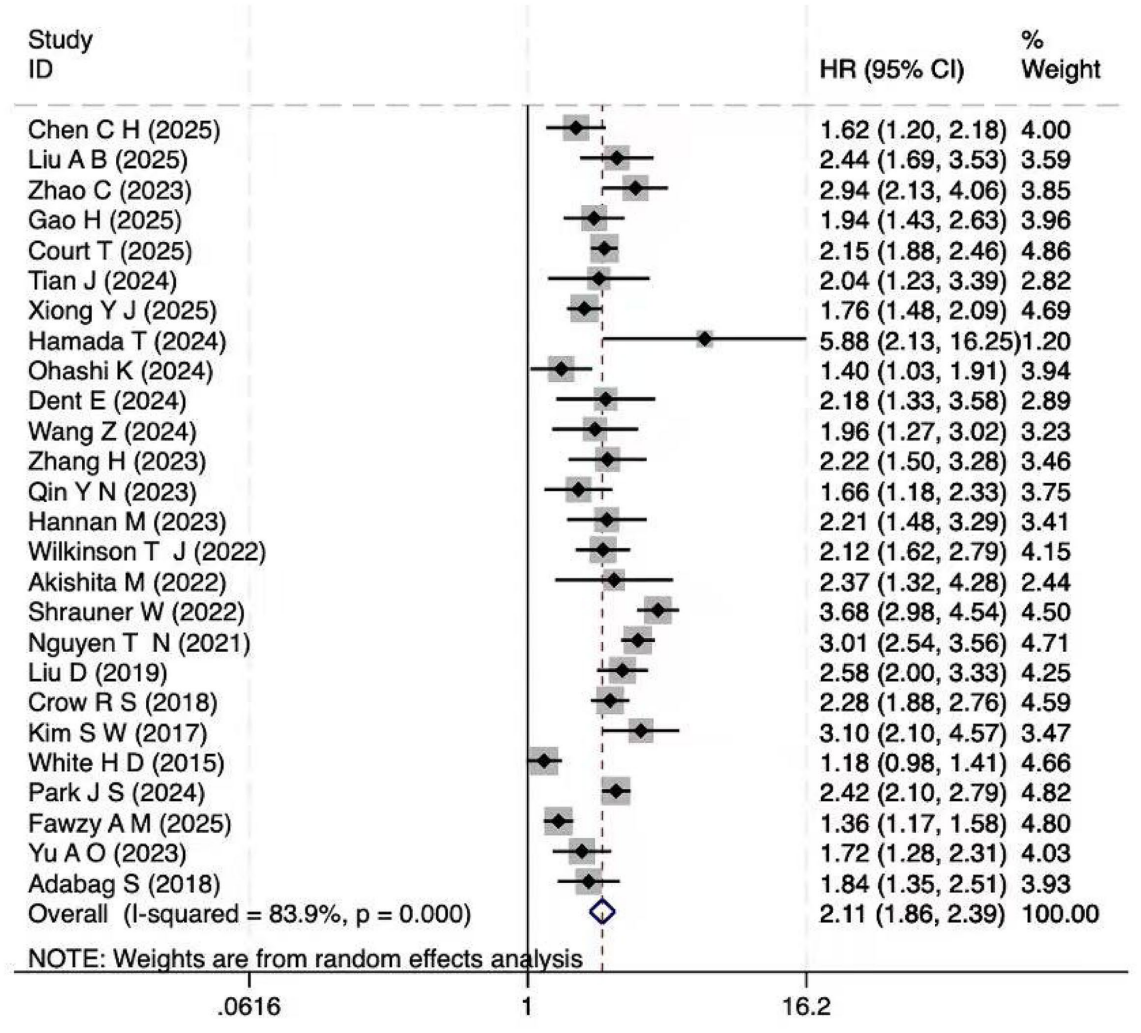
Forest plot of the HR for cardiovascular mortality associated with frailty. Pooled hazard ratio (HR = 2.11, 95% CI: 1.86–2.40) with heterogeneity assessment (*I*^2^ = 83.9%).

A total of 26 cohort studies (10, 20–44) investigated the relationship between frailty and cardiovascular disease mortality. Explored the association between frailty and cardiovascular mortality. The pooled analysis revealed a significant association between frailty and increased cardiovascular mortality (HR = 2.11; 95% CI: 1.86–2.40; *I*^2^ = 83.9%, *p* < 0.001; Figure 2). Substantial heterogeneity was observed (*I*^2^ = 83.9%), likely reflecting methodological and clinical variability across studies, such as differences in frailty definitions, populations, and follow-up durations. However, extensive subgroup and meta-regression analyses did not identify a single dominant source, and sensitivity analyses confirmed that the overall findings were robust (Supplementary Figure S1). Results for pre-frailty, which represent an intermediate stage between robustness and frailty, are presented in the subsequent subgroup analyses (Table 3).

**Table 3:** Subgroup analysis.

Subgroup	Sample size (*n*)	HR (95% CI)	*I*^2^ (%)	*p*-value
Region				
United States	11	2.30 (1.94, 2.71)	73.2	<0.001
Asians	8	2.11 (1.71, 2.61)	67.2	<0.001
Europeans	4	1.8 (1.39, 2.34)	86.4	<0.001
Pre-frailty				
Pre-frailty	8	1.80 (1.46, 2.23)	82.9	<0.001
Frailty	8	3.13 (2.26, 4.34)	75.9	<0.001

Subgroup analyses of frailty’s link to cardiovascular mortality, categorized by region and pre-frailty.

### 3.5 Subgroup analysis

Prespecified subgroup analyses confirmed the robustness of the main findings across regions, with consistent associations observed in studies conducted in North America, Europe, and Asia. Sex- and disease-specific stratifications were each based on a limited number of studies, which constrained statistical power; therefore, these results are summarized in Supplementary Table S2. Importantly, pre-frailty was also significantly associated with cardiovascular mortality (HR = 1.80; 95% CI: 1.46–2.23; *I*^2^ = 82.9%, *p* < 0.001), underscoring its prognostic relevance as an intermediate stage between robustness and frailty. Although substantial heterogeneity was observed among the eight studies included, further subgroup analyses suggested that heterogeneity was markedly reduced in studies with longer follow-up (>5 years) and in cohorts with an average age above 75 years. Detailed results of these exploratory analyses are presented in Supplementary Table S3. Beyond pre-frailty, exploratory analyses were performed to further investigate sources of heterogeneity in the overall frailty–cardiovascular mortality association. No single moderator fully explained the between-study variability, but several consistent patterns emerged. Studies with longer follow-up (≥5 years) and cohorts of very old adults (≥80 years) showed lower heterogeneity, while community-based cohorts also tended to yield more homogeneous results compared with disease-specific cohorts. In contrast, heterogeneity remained high when stratified by frailty assessment method (phenotype-based vs. deficit accumulation) or by study design (prospective vs. retrospective). Similarly, alternative cardiovascular mortality definitions yielded variable heterogeneity levels, with narrower definitions of heart disease producing more stable estimates than broader definitions including stroke or peripheral vascular disease. Meta-regression with age and follow-up as continuous moderators did not identify statistically significant associations, although effect sizes remained directionally consistent across strata (Supplementary Document S1; Supplementary Table S4).

### 3.6 Publication Bias

**Figure 3:**
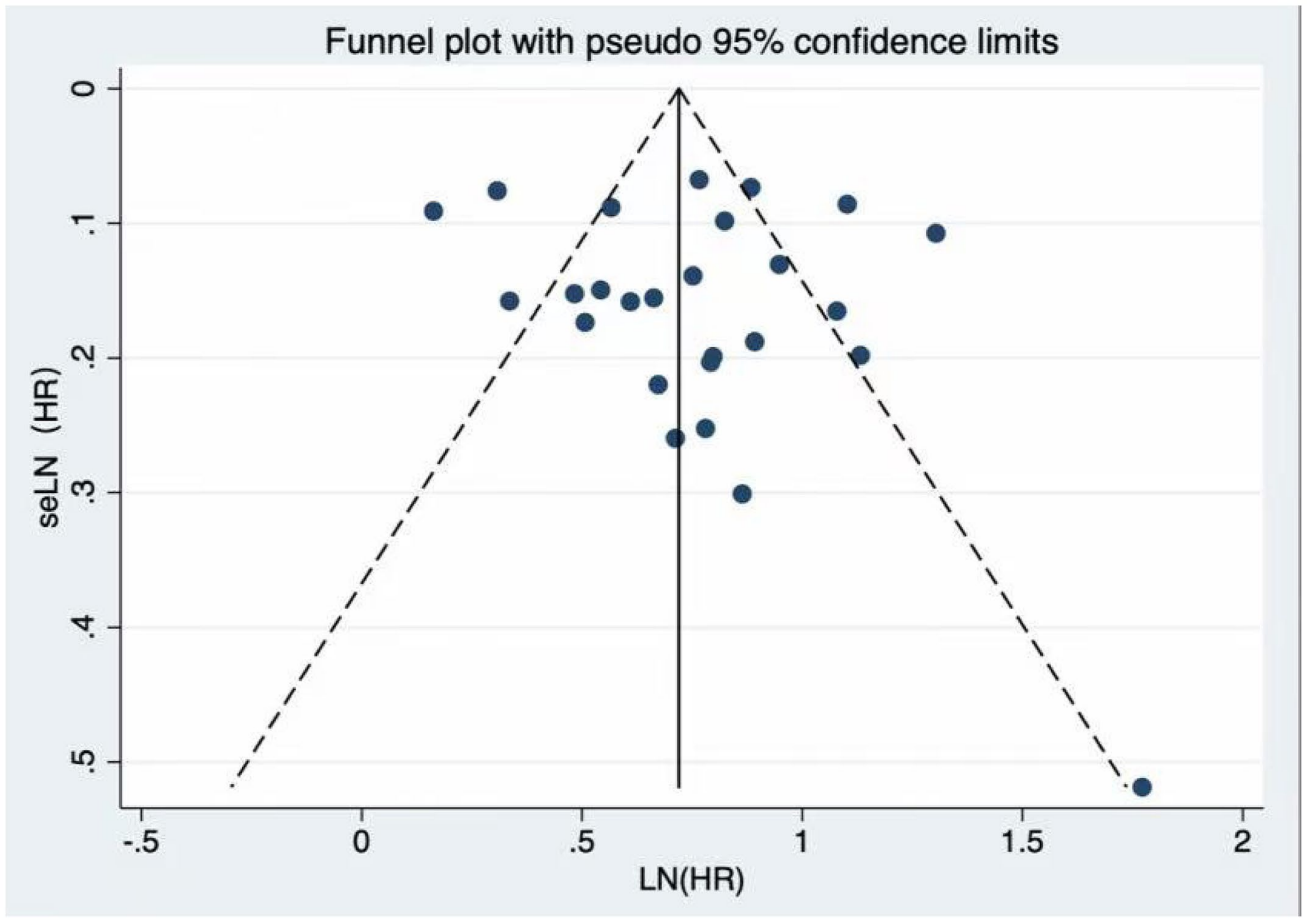
Funnel plot for publication bias. The funnel plot and Egger’s test (*p* = 0.523) showed no significant publication bias among the included studies.

Visual inspection of the funnel plot did not reveal any significant evidence of publication bias concerning frailty and cardiovascular mortality. Additionally, Egger’s regression test (*p* = 0.523) indicated no publication bias in the meta-analysis (Figure 3). Nonetheless, as in any meta-analysis, the possibility of minor undetected bias cannot be completely excluded, although the included studies covered a wide range of sample sizes and Egger’s test did not suggest a small-study effect.

## 4 Discussion

### 4.1 Main findings

This meta-analysis of 26 cohort studies, encompassing more than 4 million participants, provides robust evidence that frailty is a strong and independent predictor of cardiovascular mortality. Importantly, the analysis also revealed that individuals in the pre-frail stage—an earlier and potentially reversible condition—already carry a significantly elevated risk of cardiovascular death. This finding underscores that vulnerability to cardiovascular mortality develops well before overt frailty is established, highlighting pre-frailty as a critical window for early detection and intervention.

### 4.2 Interpretation of findings

The association between frailty and cardiovascular mortality is likely driven by several interrelated biological and clinical mechanisms that directly compromise cardiovascular health. Frailty entails multisystem decline, including sarcopenia, immune dysregulation, chronic low-grade inflammation, and impaired neuroendocrine responses, all of which accelerate atherosclerosis and predispose to fatal cardiovascular outcomes (45–48). Inflammatory activation, reflected by elevated interleukin-6 and C-reactive protein, promotes plaque instability and thrombosis, thereby contributing to sudden cardiac death and ischemic events (49, 50). Moreover, frailty is commonly accompanied by endothelial dysfunction, autonomic imbalance, malnutrition, and reduced physical activity, which diminish cardiovascular reserve and increase susceptibility to arrhythmias, hemodynamic collapse, and heart-failure–related mortality (51–54). Altered pharmacokinetics and pharmacodynamics in frail patients further increase vulnerability to under treatment or adverse drug responses, thereby worsening cardiovascular prognosis (55–57). Beyond these systemic mechanisms, accumulating evidence also suggests more direct cardiovascular pathways: elevated inflammatory biomarkers such as IL-6 and hs-CRP are strongly associated with both frailty and major adverse cardiovascular events (58–60). In addition, frailty frequently coexists with elevated cardiac stress biomarkers (e.g., NT-proBNP) (61), which are well-established predictors of cardiovascular mortality (62, 63), thereby supporting the plausibility of a biological continuum linking frailty with cardiovascular-specific mortality. Importantly, our analysis demonstrated that pre-frailty already confers a significantly elevated risk of cardiovascular mortality, likely reflecting subclinical cardiovascular abnormalities and modifiable vulnerabilities such as inactivity and poor nutrition. This underscores the importance of recognizing pre-frailty as an early at-risk state and provides a strong rationale for integrating pre-frailty into cardiovascular risk stratification and for its consideration in clinical and public health strategies.

### 4.3 Comparison with previous meta-analyses

Earlier meta-analyses (10–13) mainly examined specific groups such as acute coronary syndrome, heart failure, or dialysis patients, and focused on all-cause mortality rather than cardiovascular mortality. Our study addresses this gap by evaluating cardiovascular mortality as the primary endpoint across 26 cohorts involving community-dwelling adults, patients with cardiovascular diseases, and individuals with other chronic conditions. More recent analyses (14, 15) considered frailty and pre-frailty but were restricted to diabetes or community samples, again emphasizing all-cause mortality. In contrast, our results show that pre-frailty is already associated with a significantly increased risk of cardiovascular mortality (HR = 1.80), approaching the risk seen in frailty (HR = 2.11), suggesting that pre-frailty may represent an overlooked high-risk state. Subgroup analyses indicated similar trends in heart failure and atrial fibrillation, although the small number of studies warrants caution. The inclusion of recent East Asian cohorts (China, Japan, and South Korea) also enhances the external validity of our findings beyond Western populations. Unlike prior studies that applied a binary frailty definition (10, 11) our three-tier classification (frail, pre-frail, non-frail) enables earlier risk detection and, together with cohort evidence, provides a more comprehensive assessment of frailty’s prognostic value for cardiovascular mortality.

### 4.4 Limitations

This study has several limitations. First, substantial heterogeneity was observed, reflecting differences in frailty definitions, outcome classifications, and population types. Although extensive subgroup and meta-regression analyses were conducted (Supplementary Document S1; Supplementary Table S4), no single factor explained the variability, underscoring the need for harmonization in future research. Second, confounder adjustment was inconsistent: while most studies reported adjusted HRs, the type and number of covariates varied considerably, precluding stratification by adjustment level and leaving the possibility of residual confounding. Nevertheless, sensitivity analyses confirmed the robustness of the pooled estimates. Third, all included studies were observational, which restricts causal inference. Accordingly, the certainty of evidence would be rated low under the GRADE framework, highlighting the need for large, prospective studies. Future studies using Mendelian randomization may further strengthen causal inference. Fourth, absolute event numbers were inconsistently reported, so only relative rather than absolute risk estimates could be synthesized. Finally, potential overlap in large cohorts (e.g., NHANES) may exist, which could reduce the extent of novelty. Moreover, definitions of cardiovascular mortality varied across studies — some adopted narrow cardiac-specific endpoints (e.g., AMI, SCD, or heart failure death), while others used broader ICD-based or adjudicated definitions including stroke or peripheral vascular disease. These discrepancies may have contributed to between-study heterogeneity. In addition, the predominance of high-income cohorts may limit the generalizability of findings to low- and middle-income settings.

### 4.5 Clinical implications

Frailty is a strong and independent predictor of cardiovascular mortality, underscoring the need for routine screening, particularly in older adults. Early detection—including recognition of pre-frailty—provides an opportunity for timely interventions such as exercise, nutritional support, and rehabilitation that may prevent progression and reduce deaths. Across the included studies, both cumulative-deficit indices and phenotype-based categorical definitions were used. While cumulative approaches are comprehensive, they are often burdensome for routine care. Phenotype-based tools, by contrast, are simpler and showed broadly consistent risk estimates in our analyses, supporting their practicality. Minor differences cannot be excluded, highlighting the importance of future efforts to refine and harmonize frailty assessment for clinical use. From a broader perspective, integrating frailty assessment into cardiovascular risk stratification could improve resource allocation, identify high-risk populations, and guide preventive strategies. While current evidence does not yet support direct incorporation of frailty indices into established cardiovascular risk models (e.g., ASCVD, CHA₂DS₂-VASc), future studies should evaluate their incremental predictive value and feasibility for clinical integration once standardized assessment tools are established. Future research should standardize assessment methods, validate their feasibility in diverse settings, and rigorously evaluate interventions—such as resistance training, anti-inflammatory therapies, and personalized nutrition—for their potential to reduce cardiovascular mortality (64–68).

### 4.6 Conclusion

This meta-analysis indicates that frailty is consistently associated with increased cardiovascular mortality across diverse populations, while even pre-frailty confers a significantly elevated risk. These findings highlight pre-frailty as an under-recognized but clinically relevant stage, underscoring the value of early identification. Nevertheless, as all included studies were observational and of moderate quality, with substantial heterogeneity, the results should be interpreted with caution and not as evidence of causality. Future research should be dedicated to developing standardized and clinically practical frailty assessment tools, and to conducting large-scale prospective studies and intervention trials to determine whether modifying frailty or pre-frailty can reduce cardiovascular deaths.

## Data Availability

The original contributions presented in the study are included in the article/Supplementary material, further inquiries can be directed to the corresponding author.
