# Supplementary material for: Association of frailty and pre-frailty with cardiovascular mortality: a meta-analysis of 26 cohort studies
Source: Front Public Health. 2025 Nov 13;13:1688014. doi: 10.3389/fpubh.2025.1688014 (PMC12658331; doi:10.3389/fpubh.2025.1688014)
Supplement: Supplementary file 1 [file Data_Sheet_1.docx]

**Supplementary Results** **of Meta-Regression**

**Supplementary Result 1. Meta-regression of follow-up duration.**

**. metareg lnhr years, wsse(selnhr) knapphartung**

Meta-regression Number of obs = 26

REML estimate of between-study variance tau2 = .07012

% residual variation due to heterogeneity I-squared_res = 84.30%

Proportion of between-study variance explained Adj R-squared = -4.38%

With Knapp-Hartung modification

| lnhr | Coefficient | Std. err. | t | P>\|t\| | [95% conf. interval] | |
| --- | --- | --- | --- | --- | --- | --- |
| years | .0041106 | .0128857 | 0.32 | 0.752 | -.0224842 | .0307054 |
| _cons | .7187018 | .1018972 | 7.05 | 0.000 | .5083964 | .9290073 |

**<2 / 2–5 / ≥5 years**

**. metareg lnhr years, wsse(selnhr) bsest(reml)**

Meta-regression Number of obs = 26

REML estimate of between-study variance tau2 = .06021

% residual variation due to heterogeneity I-squared_res = 80.29%

Proportion of between-study variance explained Adj R-squared = 10.36%

With Knapp-Hartung modification

| lnhr | Coefficient | Std. err. | t | P>\|t\| | [95% conf. interval] | |
| --- | --- | --- | --- | --- | --- | --- |
| years | .1154954 | .07614 | 1.52 | 0.142 | -.0416499 | .2726407 |
| _cons | .4715909 | .1881413 | 2.51 | 0.019 | .0832863 | .8598954 |

**<5 / 5–10 / ≥10 years**

**. metareg lnhr years, wsse(selnhr) bsest(reml)**

Meta-regression Number of obs = 26

REML estimate of between-study variance tau2 = .06985

% residual variation due to heterogeneity I-squared_res = 84.33%

Proportion of between-study variance explained Adj R-squared = -3.99%

With Knapp-Hartung modification

| lnhr | Coefficient | Std. err. | t | P>\|t\| | [95% conf. interval] | |
| --- | --- | --- | --- | --- | --- | --- |
| years | .0286928 | .0833671 | 0.34 | 0.734 | -.1433684 | .2007539 |
| _cons | .6964058 | .1529621 | 4.55 | 0.000 | .3807076 | 1.012104 |

**≤2 / 2-5 / 5-10 / >10 years**

**. metareg lnhr years, wsse(selnhr) bsest(reml)**

Meta-regression Number of obs = 26

REML estimate of between-study variance tau2 = .06833

% residual variation due to heterogeneity I-squared_res = 83.55%

Proportion of between-study variance explained Adj R-squared = -1.72%

With Knapp-Hartung modification

| lnhr | Coefficient | Std. err. | t | P>\|t\| | [95% conf. interval] | |
| --- | --- | --- | --- | --- | --- | --- |
| years | .0319276 | .0566923 | 0.56 | 0.579 | -.0850795 | .1489346 |
| _cons | .6680746 | .1485181 | 4.50 | 0.000 | .3615483 | .974601 |

**Supplementary Result 2. Meta-regression of age.**

**·metareg lnhr age, wsse(selnhr) knapphartung**

Meta-regression Number of obs = 26

REML estimate of between-study variance tau2 = .07044

% residual variation due to heterogeneity I-squared_res = 84.34%

Proportion of between-study variance explained Adj R-squared = -4.86%

With Knapp-Hartung modification

| lnhr | Coefficient | Std. err. | t | P>\|t\| | [95% conf. interval] | |
| --- | --- | --- | --- | --- | --- | --- |
| age | -.0011466 | .0072336 | -0.16 | 0.875 | -.016076 | .0137828 |
| _cons | .8261574 | .5170766 | 1.60 | 0.123 | -.2410362 | 1.893351 |

**<65 / 65–74 / ≥75 y**

**. metareg lnhr age, wsse(selnhr) bsest(reml)**

Meta-regression Number of obs = 26

REML estimate of between-study variance tau2 = .07028

% residual variation due to heterogeneity I-squared_res = 84.41%

Proportion of between-study variance explained Adj R-squared = -4.62%

With Knapp-Hartung modification

| lnhr | Coefficient | Std. err. | t | P>\|t\| | [95% conf. interval] | |
| --- | --- | --- | --- | --- | --- | --- |
| age | -.017564 | .0841043 | -0.21 | 0.836 | -.1911467 | .1560187 |
| _cons | .7843192 | .1990915 | 3.94 | 0.001 | .3734145 | 1.195224 |

**<70 / 70–80 / ≥80 y**

**. metareg lnhr age, wsse(selnhr) bsest(reml)**

Meta-regression Number of obs = 26

REML estimate of between-study variance tau2 = .06752

% residual variation due to heterogeneity I-squared_res = 83.24%

Proportion of between-study variance explained Adj R-squared = -0.51%

With Knapp-Hartung modification

| lnhr | Coefficient | Std. err. | t | P>\|t\| | [95% conf. interval] | |
| --- | --- | --- | --- | --- | --- | --- |
| age | -.0516677 | .0854391 | -0.60 | 0.551 | -.2280054 | .12467 |
| _cons | .832336 | .1574448 | 5.29 | 0.000 | .5073858 | 1.157286 |

**Supplementary Result 3. Meta-regression of disease characteristics of the population.**

**1 None /2 cardiac disease /3 metabolism and dialysis**

metareg lnhr disease, wsse(selnhr) bsest(reml)

Meta-regression Number of obs = 26

REML estimate of between-study variance tau2 = .06859

% residual variation due to heterogeneity I-squared_res = 84.26%

Proportion of between-study variance explained Adj R-squared = -2.11%

With Knapp-Hartung modification

| lnhr | Coefficient | Std. err. | t | P>\|t\| | [95% conf. interval] | |
| --- | --- | --- | --- | --- | --- | --- |
| disease | -.0510526 | .0754896 | -0.68 | 0.505 | -.2068555 | .1047503 |
| _cons | .8369572 | .1495072 | 5.60 | 0.000 | .5283895 | 1.145525 |

**Supplementary Result 4. Meta-regression of methods for constructing or classifying frailty index (classification / summation).**

metareg lnhr frailty, wsse(selnhr) bsest(reml)

Meta-regression Number of obs = 26

REML estimate of between-study variance tau2 = .05518

% residual variation due to heterogeneity I-squared_res = 80.80%

Proportion of between-study variance explained Adj R-squared = 17.85%

With Knapp-Hartung modification

| lnhr | Coefficient | Std. err. | t | P>\|t\| | [95% conf. interval] | |
| --- | --- | --- | --- | --- | --- | --- |
| Frailty | .225493 | .1118323 | 2.02 | 0.055 | -.0053175 | .4563036 |
| _cons | .4103066 | .1739194 | 2.36 | 0.027 | .0513545 | .7692587 |

**Supplementary Result 5. Meta-regression of study type (prospective cohort and retrospective cohort).**

**. metareg lnhr studytype, wsse(selnhr) bsest(reml)**

Meta-regression Number of obs = 26

REML estimate of between-study variance tau2 = .0665

% residual variation due to heterogeneity I-squared_res = 84.42%

Proportion of between-study variance explained Adj R-squared = 1.00%

With Knapp-Hartung modification

| lnhr | Coefficient | Std. err. | t | P>\|t\| | [95% conf. interval] | |
| --- | --- | --- | --- | --- | --- | --- |
| Study type | .1191887 | .1236814 | 0.96 | 0.345 | -.1360771 | .3744545 |
| _cons | .5503835 | .2098418 | 2.62 | 0.015 | .1172913 | .9834757 |

**Supplementary Result 6. Meta-regression of definition of mortality.**

0.NR (Not Reported) : The definition is not reported.

1. Narrow: only direct cardiac causes (AMI/MI, SCD, malignant arrhythmia, heart failure death, etc.).

2. Intermediate: heart disease (coronary heart disease, heart failure, hypertensive heart disease) ± some circulatory diseases (e.g. I00-i09, I11, I13, I20-I51), usually without stroke or peripheral vessels.

3. Broad: almost the entire cardiovascular system (ICD-10 I00-I99), including stroke, peripheral vascular disease, etc.

**Group 0-1-2-3**

metareg lnhr mortality, wsse(selnhr) bsest(reml)

Meta-regression Number of obs = 26

REML estimate of between-study variance tau2 = .06874

% residual variation due to heterogeneity I-squared_res = 83.71%

Proportion of between-study variance explained Adj R-squared = -2.34%

With Knapp-Hartung modification

| lnhr | Coefficient | Std. err. | t | P>\|t\| | [95% conf. interval] | |
| --- | --- | --- | --- | --- | --- | --- |
| mortality | -.0454519 | .0600087 | -0.76 | 0.456 | -.1693038 | .0784 |
| _cons | .8353793 | .1343819 | 6.22 | 0.000 | .5580287 | 1.11273 |

**Group 1-2-3**

metareg lnhr mortality, wsse(selnhr) bsest(reml)

Meta-regression Number of obs = 23

REML estimate of between-study variance tau2 = .07264

% residual variation due to heterogeneity I-squared_res = 84.25%

Proportion of between-study variance explained Adj R-squared = -5.94%

With Knapp-Hartung modification

| lnhr | Coefficient | Std. err. | t | P>\|t\| | [95% conf. interval] | |
| --- | --- | --- | --- | --- | --- | --- |
| mortality | -.002024 | .0865635 | -0.02 | 0.982 | -.1820427 | .1779947 |
| _cons | .726916 | .2057936 | 3.53 | 0.002 | .2989447 | 1.154887 |
