# Supplementary material for: Association of frailty and pre-frailty with cardiovascular mortality: a meta-analysis of 26 cohort studies
Source: Front Public Health. 2025 Nov 13;13:1688014. doi: 10.3389/fpubh.2025.1688014 (PMC12658331; doi:10.3389/fpubh.2025.1688014)

**Supplementary Table 1**

**1.1** **Search strategy for PubMed**

|  | | | | | |
| --- | --- | --- | --- | --- | --- |
| Search number | Query | Sort By | Search Details | Results | Date |
| 8 | ((cardiovascular death[Title/Abstract]) OR (cardiovascular mortality[Title/Abstract])) AND (((((((Frailty[Title/Abstract]) OR (Frailties[Title/Abstract])) OR (Frailness[Title/Abstract])) OR (Frailty Syndrome[Title/Abstract])) OR (Debility[Title/Abstract])) OR (Debilities[Title/Abstract])) OR ("Frailty"[Mesh])) | Most Recent | ("cardiovascular death"[Title/Abstract] OR "cardiovascular mortality"[Title/Abstract]) AND ("Frailty"[Title/Abstract] OR "Frailties"[Title/Abstract] OR "Frailness"[Title/Abstract] OR "frailty syndrome"[Title/Abstract] OR "Debility"[Title/Abstract] OR "Debilities"[Title/Abstract] OR "Frailty"[MeSH Terms]) | 196 | 2025/7/18 |
| 7 | (((cardiovascular death[Title/Abstract]) OR (cardiovascular mortality[Title/Abstract])) OR ("Mortality"[Mesh])) AND (((((((Frailty[Title/Abstract]) OR (Frailties[Title/Abstract])) OR (Frailness[Title/Abstract])) OR (Frailty Syndrome[Title/Abstract])) OR (Debility[Title/Abstract])) OR (Debilities[Title/Abstract])) OR ("Frailty"[Mesh])) | Most Recent | ("cardiovascular death"[Title/Abstract] OR "cardiovascular mortality"[Title/Abstract] OR "Mortality"[MeSH Terms]) AND ("Frailty"[Title/Abstract] OR "Frailties"[Title/Abstract] OR "Frailness"[Title/Abstract] OR "frailty syndrome"[Title/Abstract] OR "Debility"[Title/Abstract] OR "Debilities"[Title/Abstract] OR "Frailty"[MeSH Terms]) | 2,082 | 2025/7/18 |
| 6 | ((cardiovascular death[Title/Abstract]) OR (cardiovascular mortality[Title/Abstract])) OR ("Mortality"[Mesh]) | Most Recent | "cardiovascular death"[Title/Abstract] OR "cardiovascular mortality"[Title/Abstract] OR "Mortality"[MeSH Terms] | 467,938 | 2025/7/18 |
| 5 | (cardiovascular death[Title/Abstract]) OR (cardiovascular mortality[Title/Abstract]) | Most Recent | "cardiovascular death"[Title/Abstract] OR "cardiovascular mortality"[Title/Abstract] | 29,906 | 2025/7/18 |
| 4 | "Mortality"[Mesh] | Most Recent | "Mortality"[MeSH Terms] | 442,918 | 2025/7/18 |
| 3 | ((((((Frailty[Title/Abstract]) OR (Frailties[Title/Abstract])) OR (Frailness[Title/Abstract])) OR (Frailty Syndrome[Title/Abstract])) OR (Debility[Title/Abstract])) OR (Debilities[Title/Abstract])) OR ("Frailty"[Mesh]) | Most Recent | "Frailty"[Title/Abstract] OR "Frailties"[Title/Abstract] OR "Frailness"[Title/Abstract] OR "frailty syndrome"[Title/Abstract] OR "Debility"[Title/Abstract] OR "Debilities"[Title/Abstract] OR "Frailty"[MeSH Terms] | 36,198 | 2025/7/18 |
| 2 | (((((Frailty[Title/Abstract]) OR (Frailties[Title/Abstract])) OR (Frailness[Title/Abstract])) OR (Frailty Syndrome[Title/Abstract])) OR (Debility[Title/Abstract])) OR (Debilities[Title/Abstract]) | Most Recent | "Frailty"[Title/Abstract] OR "Frailties"[Title/Abstract] OR "Frailness"[Title/Abstract] OR "frailty syndrome"[Title/Abstract] OR "Debility"[Title/Abstract] OR "Debilities"[Title/Abstract] | 35,570 | 2025/7/18 |
| 1 | "Frailty"[Mesh] | Most Recent | "Frailty"[MeSH Terms] | 13,517 | 2025/7/18 |

**1.2 Search strategy for Embase**

| No. | Query | Results | Date |
| --- | --- | --- | --- |
| #5 | #2 AND #4 | 334 | 18-Jul-25 |
| #4 | #1 OR #3 | 59105 | 18-Jul-25 |
| #3 | frailties:ti,ab OR frailness:ti,ab OR 'frailty syndrome':ti,ab OR debility:ti,ab OR debilities:ti,ab OR 'frailty':ti,ab | 52763 | 18-Jul-25 |
| #2 | 'cardiovascular death':ab,ti OR 'cardiovascular mortality':ab,ti | 48239 | 18-Jul-25 |
| #1 | 'frailty'/exp | 40738 | 18-Jul-25 |

**1.3 Search strategy for cochrane**

| No. | Query | Results | Date |
| --- | --- | --- | --- |
| #9 | #5 AND #6 | 235 | 18-Jul-25 |
| #8 | #3 AND #7 | 27 | 18-Jul-25 |
| #7 | 'cardiovascular death':ti OR 'cardiovascular mortality':ti | 5982 | 18-Jul-25 |
| #6 | 'cardiovascular death':ab,ti OR 'cardiovascular mortality':ab,ti | 48239 | 18-Jul-25 |
| #5 | #1 OR #4 | 43127 | 18-Jul-25 |
| #4 | frailties:ti,ab OR frailness:ti,ab OR 'frailty syndrome':ti,ab OR debility:ti,ab OR debilities:ti,ab | 3475 | 18-Jul-25 |
| #3 | #1 OR #2 | 41032 | 18-Jul-25 |
| #2 | frailties:ti OR frailness:ti OR 'frailty syndrome':ti OR debility:ti OR debilities:ti | 674 | 18-Jul-25 |
| #1 | 'frailty'/exp | 40738 | 18-Jul-25 |

**Supplementary Figure A**

**Sensitivity analysis**


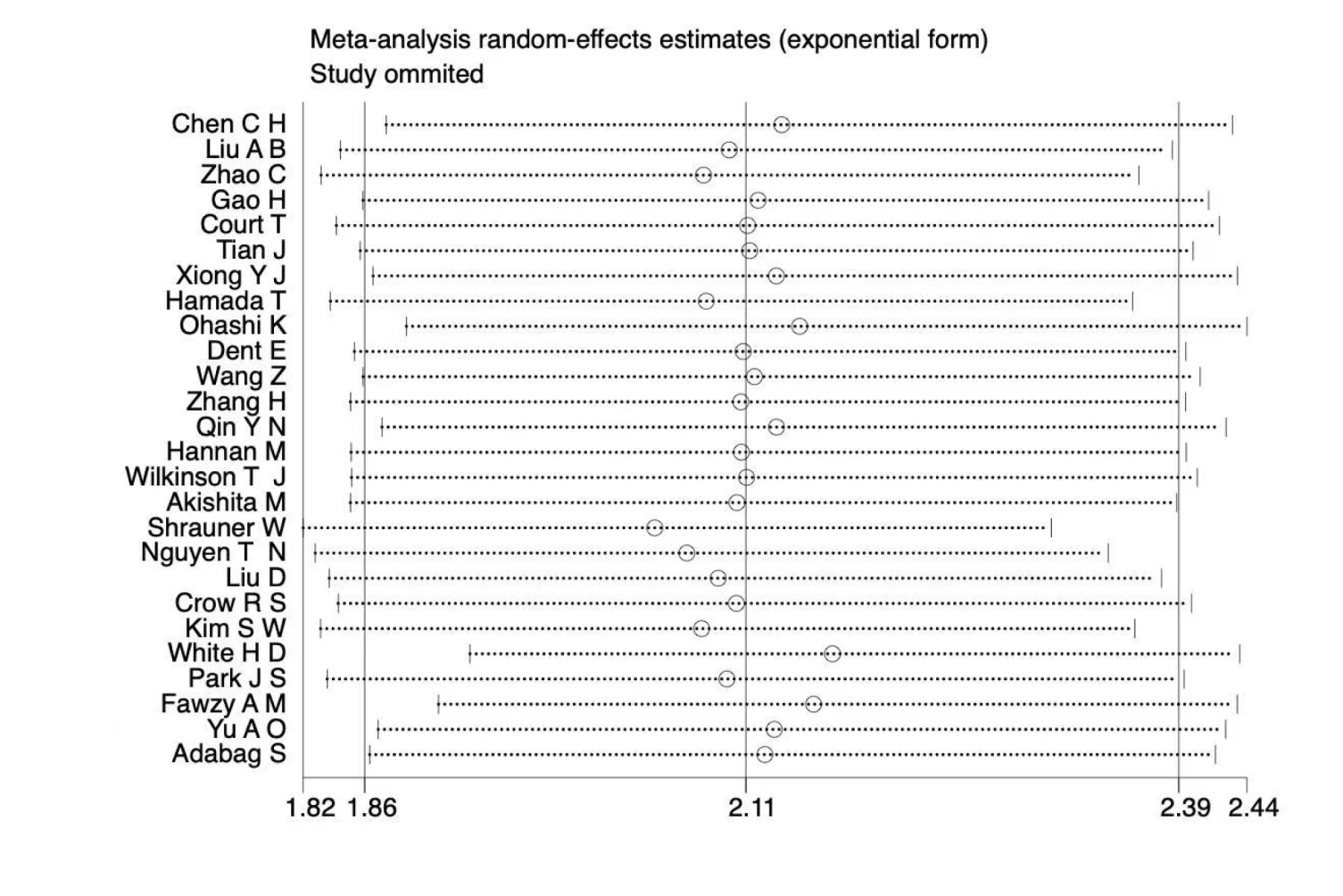

Supplement: Supplementary file 2 [file Supplementary_file_1.docx]
