# Supplementary material for: Association of frailty and pre-frailty with cardiovascular mortality: a meta-analysis of 26 cohort studies
Source: Front Public Health. 2025 Nov 13;13:1688014. doi: 10.3389/fpubh.2025.1688014 (PMC12658331; doi:10.3389/fpubh.2025.1688014)
Supplement: Supplementary file 3 [file Supplementary_file_2.docx]

**Supplementary Table 2**

**Supplement Table 2.1 Subgroup analysis of Sex**

| **Sex** | **Sample size(n)** | **HR(95%CI)** | **I²(%)** | ***P*-value** |
| --- | --- | --- | --- | --- |
| **Male** | 2 | 1.38 (1.10, 1.72) | 3.3 | 0.309 |
| **Female** | 3 | 1.73 (1.15, 2.61) | 51.8 | 0.126 |

**Supplement Table 2.2 Subgroup analysis of Disease characteristics**

| **disease**  **characteristics** | **Sample size(n)** | **HR(95%CI)** | **I²(%)** | ***P*-value** |
| --- | --- | --- | --- | --- |
| **AF** | 4 | 2.17 (1.44, 3.27) | 91.9 | < 0.001 |
| **HF** | 3 | 2.17 (1.16, 4.04) | 74.1 | 0.021 |
| **MI/UA** | 2 | 1.40 (0.97, 2.02) | 78.7 | 0.03 |
| **DM** | 3 | 2.09 (1.39, 3.14) | 91 | < 0.001 |
| **CKD** | 2 | 2.10 (1.57, 2.80) | 0 | 0.705 |

*Notes: diabetes mellitus (DM), heart failure (HF), atrial fibrillation (AF), Chronic Kidney Disease (CKD), myocardial infarction (MI),* *Unstable angina pectoris (UA)*

**Supplementary Table 3**

**Subgroup analysis of follow-up duration and mean age in pre-frailty population.**

| **Pre-frailty** | **Sample size(n)** | **HR(95%CI)** | **I²(%)** | ***P*-value** |
| --- | --- | --- | --- | --- |
| **Follow-up duration** |  |  |  |  |
| **<5y** | 4 | 1.46 (1.10,1.94) | 72.5 | 0.012 |
| **≥5y** | 4 | 2.19 (1.84, 2.61) | 44.5 | 0.144 |
| **Mean age** |  |  |  |  |
| **<75y** | 5 | 1.86 (1.33, 2.59) | 89.3 | 0.000 |
| **≥75y** | 3 | 1.69 (1.53, 1.87) | 0.0 | 0.948 |

**Supplementary Table 4**

**Supplement Table 4.1 Subgroup analysis of follow-up duration.**

| **Follow-up duration** | **Sample size(n)** | **HR(95%CI)** | **I²(%)** | ***P*-value** |
| --- | --- | --- | --- | --- |
| **Group 1** |  |  |  |  |
| **<2y** | 4 | 1.63 (1.20, 2.22) | 86.2 | 0.000 |
| **2–5y** | 9 | 2.28 (1.75,2.98) | 82.8 | 0.000 |
| **≥5y** | 13 | 2.18 (2.01,2.37) | 29.8 | 0.146 |
| **Group 2** |  |  |  |  |
| **<5y** | 13 | 1.90 (1.77, 2.04) | 90.8 | 0.000 |
| **5–10y** | 9 | 2.30 (2.12, 2.50) | 15.4 | 0.305 |
| **≥10y** | 4 | 2.02 (1.83, 2.23) | 15.9 | 0.312 |
| **Group 3** |  |  |  |  |
| **≤2y** | 8 | 2.07 (1.45, 2.95) | 92.6 | 0.000 |
| **2-5y** | 5 | 2.11 (1.58,2.80) | 76.1 | 0.002 |
| **5-10y** | 9 | 2.29 (2.09,2.52) | 15.4 | 0.305 |
| **>10y** | 4 | 2.01 (1.79, 2.26) | 15.9 | 0.312 |

**Supplement Table 4.2 Subgroup analysis of age.**

| **Age** | **Sample size(n)** | **HR(95%CI)** | **I²(%)** | ***P*-value** |
| --- | --- | --- | --- | --- |
| **Group 1** |  |  |  |  |
| **<65y** | 4 | 2.15 (1.78,2.60) | 63.8 | 0.041 |
| **65–74y** | 10 | 2.08 (1.68,2.58) | 87.2 | 0.000 |
| **≥75y** | 12 | 2.13(1.68,2.69) | 85.1 | 0.000 |
| **Group 2** |  |  |  |  |
| **<70y** | 11 | 2.20 (1.93,2.51) | 68.1 | 0.001 |
| **70–80y** | 10 | 2.07 (1.60,2.67) | 91.1 | 0.000 |
| **≥80y** | 5 | 1.98 (1.47, 2.67) | 55.3 | 0.062 |

**Supplement Table 4.3 Subgroup analysis of disease characteristics of the population.**

| **Disease characteristics of the population** | **Sample size(n)** | **HR(95%CI)** | **I²(%)** | ***P*-value** |
| --- | --- | --- | --- | --- |
| **None** | 11 | 2.33 (2.16,2.51) | 60.1 | 0.005 |
| **cardiac disease** | 9 | 1.70 (1.57, 1.84) | 87.9 | 0.000 |
| **metabolism and dialysis** | 6 | 2.21 (2.01,2.44) | 83.0 | 0.000 |

**Supplement Table 4.4 Subgroup analysis of methods for constructing or classifying frailty index.**

| **Methods for constructing or classifying frailty index** | **Sample size(n)** | **HR(95%CI)** | **I²(%)** | ***P*-value** |
| --- | --- | --- | --- | --- |
| **classification** | 14 | 1.81 (1.69, 1.93) | 83.0 | 0.000 |
| **summation** | 12 | 2.36 (2.21,2.53) | 77.3 | 0.000 |

**Supplement Table 4.5 Subgroup analysis of definition of mortality.**

| **Definition of mortality** | **Sample size(n)** | **HR(95%CI)** | **I²(%)** | ***P*-value** |
| --- | --- | --- | --- | --- |
| **NR** | 3 | 2.55 (1.96,3.33) | 58.3 | 0.091 |
| **Narrow** | 5 | 1.71 (1.24, 2.35) | 77.9 | 0.001 |
| **Intermediate** | 8 | 2.59 (2.26,2.98) | 54.6 | 0.031 |
| **Broad** | 10 | 1.86 (1.61,2.16) | 69.9 | 0.000 |

*NR (Not Reported) : The definition is not reported. Narrow: only direct cardiac causes (AMI/MI, SCD, malignant arrhythmia, heart failure death, etc.). Intermediate: heart disease (coronary heart disease, heart failure, hypertensive heart disease) ± some circulatory diseases (e.g. I00-i09, I11, I13, I20-I51), usually without stroke or peripheral vessels. Broad: almost the entire cardiovascular system (ICD-10 I00-I99), including stroke, peripheral vascular disease, etc.*
